# Vaccine Passports and Indian Country: Nothing Fast About It

**DOI:** 10.1177/00333549221094557

**Published:** 2022-06-01

**Authors:** Alec J. Calac, Aila Hoss

**Affiliations:** 1 School of Medicine, University of California, San Diego, San Diego, CA, USA; 2 School of Public Health, San Diego State University, San Diego, CA, USA; 3 Native American Law Center, University of Tulsa College of Law, Tulsa, OK, USA

**Keywords:** vaccines, American Indian or Alaska Native, health policy, law/legal issues, infectious disease

Indian Country has been substantially affected by the COVID-19 pandemic, with similar hospitalization and mortality rates observed during the 1918 and 2009 influenza pandemics.^1-4^ Before the widespread availability of COVID-19 vaccines, tribes attempted to mitigate the impact of the pandemic in their communities but experienced serious legal and policy challenges at the state and federal level.^5^ Despite these challenges, tribes have administered successful vaccination programs for their citizens and communities.^6-11^ According to September 2021 data from the Centers for Disease Control and Prevention (CDC), American Indian and Alaska Native people had the highest rates of COVID-19 vaccination by race and ethnicity (56.3% had ≥1 dose; 48.5% were fully vaccinated) in the United States.^12^

With vaccination uptake steadily increasing, many jurisdictions have started to modify or completely lift COVID-19 restrictions.^13^ The effectiveness of vaccination in preventing hospitalization and serious illness, even amid variant-specific surges, has prompted officials to explore the utility of digital vaccine passports, which allow people to quickly verify their vaccination status using information from their electronic health record (EHR).^14^ The White House recommends a self-attestation or honor system for declaring COVID-19 vaccination status.^15^ This recommendation has not stopped jurisdictions from using digital vaccine passports. Digital vaccine passports may unintentionally create barriers for patients in health care systems with limited EHR interoperability and decentralized operations, such as the Indian Health Service (IHS). This commentary explores equity issues that may arise for American Indian and Alaska Native people concerning the issuance of digital vaccine passports.

## Tribal Sovereignty and Public Health Authority

The utility and implications of vaccine passports in Indian Country are affected by the legal relationships among tribes, states, and the federal government. Tribes are sovereign nations with the inherent authority and obligation to promote public health in their communities. This authority can be in the form of public health policy making and enforcement, programmatic and community engagement, and other services based on the culture and priorities of a tribe. During the COVID-19 pandemic, for example, some tribes quickly barred nonessential travel through their communities, secured personal protective equipment, and sought relief from the federal government in line with the federal trust responsibility on which we elaborate hereinafter.^16^

As with all jurisdictions, tribes can work in partnership with other tribes, states, local governments, and the federal government to advance their public health goals. The federal government maintains treaty and trust obligations to provide health care to American Indian and Alaska Native people and to protect and support tribal lands, communities, and sovereignty.^17^ A product of colonization and genocide, the principles of federal Indian law allow the federal government to exercise concurrent jurisdiction in Indian Country. This body of law authorizes federal legislation on tribes and American Indian and Alaska Native people. In recent decades, Congress has passed more legislation to support tribal self-governance^18^; however, not all federal laws and policies potentially benefiting tribes are implemented with adequate consultation. In the context of health care, the federal government consistently reneges on its legal obligation to provide health care through chronic underfunding and mismanagement of critical agencies such as IHS.^19,20^

In addition, tribes regularly have to challenge infringement of their authority or lack of coordination by state and local governments. In general, state authority does not extend to tribal members on tribal lands, and tribes can assert jurisdiction over non-member activities with the reservation in limited circumstances. Here, too, the COVID-19 pandemic provides numerous examples. For example, South Dakota challenged tribal checkpoints put in place on the Oglala Lakota and Cheyenne River Sioux reservations to limit nonessential travel through highways on tribal lands, and nontribal members disregarded tribal COVID-19 restrictions across the country.^21,22^

## Vaccine Distribution and Tracking

The federal government purchased COVID-19 vaccines for distribution across the country through agreements between pharmaceutical companies under Operation Warp Speed. Vaccines were distributed to states, tribes, territories, and federal health programs such as the IHS and Veterans Health Administration.^23-25^ In consultation with tribal leaders, the federal government allowed for Tribal and Urban Indian health programs to receive COVID-19 vaccine allotments directly from the federal government or, if preferred, from state and county health departments.^26^

As a condition for enrolling as a state-based vaccine provider, health care providers were required to register in state immunization registries and collect and report vaccination information.^27,28^ Tribal and Urban Indian health programs opting for state-based vaccine allotments are required to log their vaccinations in state-maintained registries (eg, California Immunization Registry), but those requirements are optional for IHS and Tribal and Urban Indian health programs opting for allotments directly from the federal government.^29^ CDC collects vaccine administration data, but it does not maintain patient-specific vaccination records.^30^ Thus, while optional vaccination reporting requirements protect tribal health data, they will likely lead to regional gaps in vaccination reporting to states from a subset of Indian health facilities.^31^ As of October 2021, 356 IHS, Tribal, and Urban Indian health programs chose to receive COVID-19 vaccines directly from the federal government, representing 54.6%, or just more than half of all IHS facilities (N = 652) (Table 1). The total number of facilities by IHS Area and operator (IHS/Tribal/Urban) is not available.

**Table 1.** Indian Health Service (IHS) and Tribal and Urban Indian health facilities (IHS/Tribal/Urban) receiving federal COVID-19 vaccine allotments, October 2021^a^

IHS area	No. of facilities (IHS/Tribal/Urban) receiving COVID-19 vaccines (n = 356)
Albuquerque Area	28
Bemidji Area	36
Billings Area	20
California Area	73
Great Plains Area	25
Nashville Area	28
Navajo Area	23
Oklahoma City Area	63
Phoenix Area	34
Portland Area	23
Tucson Area	3

a As of October 2021, 356 IHS/Tribal/Urban facilities chose to receive COVID-19 vaccines directly from the federal government, representing 54.6%, or just more than half of all IHS facilities (N = 652). Data source: IHS.^32,33^

## Emerging Vaccine Passport Policies

State health departments, third-party companies, and tribes began developing vaccine passports and/or vaccination verification systems in early 2021.

New York State was the first to announce a digital vaccine passport (“Excelsior Pass”) for use at sporting events and other commercial venues. The Excelsior Pass draws from COVID-19 vaccination registries maintained by New York State and is not linked to other state or federal immunization registries. No specific mentions of COVID-19 vaccination data-sharing agreements with tribes in New York State appear on the Excelsior website.^34^

In June 2021, Ehave, Inc, announced a partnership with Health Wizz, a mobile health record application on Ethereum blockchain, launching its own version of a digital vaccine passport (“Ehave Medical Passport”) to allow individuals to voluntarily share their verified vaccination status. Their application relies on the use of Fast Healthcare Interoperability Resources (FHIR) application program interfaces.^35^ Similarly, the MITRE Corporation and a Steering Group comprising representatives from the Mayo Clinic, Microsoft, The Commons Project Foundation, Evernorth, CARIN Alliance, UC San Diego Health, and Apple developed an open-source SMART Health Card Framework allowing for states and other entities to develop vaccine verification systems.^36^ The framework has already been adopted by the California Department of Public Health, and, like the Excelsior Pass, it is linked to state vaccination registries.^37^

Selected vaccine data practices involving Indigenous governments or communities include maintaining separate vaccination registries and creating vaccination cards (Table 2). In the Pacific Northwest, the Affiliated Tribes of Northwest Indians, an organization representing 57 tribes in 6 states, adopted recommendations from the American Indian Health Commission Tribal/Urban Indian Health Immunization Coalition (TUIHIC) calling for an official COVID-19 vaccination record for American Indian and Alaska Native people. TUIHIC requests recognition of tribal vaccination records in “non-Indian, nationwide health systems and federal, state and local jurisdictions,” citing the public health authority of tribes to issue these documents in an accurate and timely manner.^43^ This recognition is unlikely to be an issue in states such as Oregon, where the state’s health authority describes proof of vaccination as a document from a “[t]ribal, federal, state, local government or health care provider,” so long as it displays the same information as the federal COVID-19 vaccination card.^41^ However, recognition of tribal vaccination cards may be an issue for states unfamiliar with tribal public health authority. Additional examples of vaccine data practices can be seen with tribes in California and Alaska and Aboriginal communities in Australia and Canada.^29,38-42^

**Table 2.** Selected data-related practices involving vaccines and Indigenous governments or communities

Jurisdiction or organization	Document/framework	Description of practice
Australian government	COVID-19 Vaccination Program Implementation Plan: Aboriginal and Torres Strait Islander Peoples	Establish and manage a system to track and trace vaccine doses that directly interact with existing systems such as the Australian Immunisation Register and clinical software.^38^
Algoma Public Health (Canada)	Indigenous Health and COVID-19	If you received your vaccine out of province or country, you can send Algoma Public Health a request to enter your COVID-19 vaccine into the Ontario database (COVax).^39^
Qemirtalek Coast Corporation (Native Village of Kongiganak, Alaska)	Not available	Sheila Phillip, Kongiganak Traditional Council Secretary, said that the vaccine passport is a way to start reopening businesses in a safe manner.^40^ “The general manager for Qemirtalek Coast Corporation, Harvey Paul, said that his store is only allowing four people in at a time. Paul said that his employees verify that a customer is vaccinated by checking that their name is on a list of vaccinated individuals that they get from the [T]ribe.”^40^
Oregon Health Authority	Interim Guidance for Fully Vaccinated Individuals	The Oregon Health Authority describes a proof of vaccination as a document issued by “a [T]ribal, federal, state, local government or health care provider” that includes the citizen’s name, date of birth, the COVID-19 jab administered, and date and place of vaccination.^41^
Indian Health Service (United States)	COVID-19 Vaccine Data Management	In addition to submitting the data to IHS, Tribal, and Urban Indian health programs may also report these data to state or local jurisdiction immunization information systems.^29^
Harrah Resort Southern California (an enterprise owned by the Rincon Band of Luiseño Indians)	Resort Health and Sanitation Plan	Face masks are required for unvaccinated guests and recommended for all guests indoors, regardless of vaccination status.^42^

## Vaccine Passports and Tribal Health Equity

Five themes emerge when considering vaccine passports through the lens of tribal health equity. First, as others have identified, technology access and compatibility can prevent access to vaccine passport systems. The lack of access is acutely felt in Indian Country, where only 65% of residents on reservations had access to fixed broadband services, according to a 2018 report.^44^ Similarly, while the FHIR standard has wide implementation across modern EHRs, it is not widely available in EHRs used at IHS facilities. Findings from the IHS Health IT Modernization Project reveal that FHIR standards are not easily implementable within the commonly used IHS Resource and Patient Management System, an outdated EHR system that IHS is working to retire. Complicating the matter, Tribal and Urban Indian health programs maintain their own instance of the IHS Resource and Patient Management System or their respective EHR, which may or may not have the capacity to use the FHIR interface.^45^ This means that there is no standardization or central collection of IHS health records to facilitate a less burdensome creation of digital vaccine records for personal use. IHS maintains the National Patient Information Reporting System (NPIRS) to collect various types of financial, environmental, and health care information from IHS, Tribal, and Urban Indian health facilities.^46^ While NPIRS collects COVID-19 vaccination data, facilities that do not use an EHR that is compatible with IHS’s central EHR system are not able to submit data to NPIRS.^47,48^

Second, tribes that implement their own vaccine passport systems may struggle with having them recognized by other governments. This difficulty can be demonstrated by challenges to tribal membership cards. The recognition of documents issued by tribes for the purposes of travel, such as a tribal membership card for use at an airport or an Iroquois passport at an international border, has precedent. However, these forms of identification have not been without controversy, with routine incidents between tribal members and Transportation Security Administration officers and long-standing concerns about the legality of the Iroquois passport.^49-51^ Similarly, state election laws that require photo identification can recognize tribal membership cards as a valid form of identification.^52^ Here, too, state elections laws can be subject to litigation when they do not recognize these forms of identification.^53-55^

Third, it is imperative that tribes have the right to access vaccination reporting systems and assert control over their own data in accordance with the principle of Indigenous Data Sovereignty.^56^ A strong example of this principle in practice can be seen with the National Institutes of Health All of Us Research Program, which released guidelines detailing the collection, ownership, and right to access biospecimen data specific to American Indian and Alaska Native participants.^57,58^ This principle ensures that such data, and any physical or digital derivatives, are managed in a way that respects the laws and cultural practices of tribal governments, and no data can be accessed or disseminated without prior authorization.^59^

Next, vaccine passport policies can lead to jurisdictional challenges that arise from such factors as the scope of the provision, the jurisdiction implementing it, and its enforcement. As discussed previously, states generally do not have jurisdiction on tribal lands, and tribes may have limited jurisdiction over non-tribal members even within the boundaries of a reservation. These relatively simple legal principles are riddled with exceptions, nuances, and ambiguities. For example, a state or local government attempting to implement and enforce a vaccine policy in tribal lands could infringe on tribal jurisdictions.

Finally, it is imperative that states establishing vaccine passport policies engage in formal, government-to-government consultation with tribes. Lack of or inadequate consultation can lead to poor health outcomes for American Indian and Alaska Native communities.^60^ Tribal consultation would ensure that COVID-19 vaccine passports or verification systems respect tribal sovereignty and the right of tribes to maintain control over their data and related health information in the digital space.

## Conclusion

American Indian and Alaska Native people may not have equitable access to digital COVID-19 vaccine records, especially those served by IHS, raising digital equity concerns with the greater US population. Tribes should not have to release their own health data to third-party companies to regain access to businesses and institutions that require proof of COVID-19 vaccination. Furthermore, it is unclear if most state health departments will accept COVID-19 vaccination records issued by tribes.
